# Assessment of the Antibiofilm Performance of Silver-Containing Wound Dressings: A Dual-Species Biofilm Model

**DOI:** 10.7759/cureus.70086

**Published:** 2024-09-24

**Authors:** Kate Meredith, Matilda M Coleborn, Lucy E Forbes, Daniel G Metcalf

**Affiliations:** 1 Research and Development, Convatec, Deeside, GBR

**Keywords:** biofilm, dressings, hard-to-heal, klebsiella pneumoniae, methicillin-resistant staphylococcus aureus, mrsa, silver, wound

## Abstract

Background

It is commonly accepted that microorganisms found within hard-to-heal wounds are present in biofilm form. Biofilms are often polymicrobial in nature, which increases their virulence and tolerance to antimicrobial agents. The aim of this study was to compare the antibiofilm activity of silver-containing antimicrobial wound dressings in a dual-species simulated wound biofilm model.

Materials and methods

Four silver-containing wound dressings were evaluated *in vitro*: Aquacel^®^ Ag+ Extra™ dressing, KerraContact^®^ Ag dressing, Durafiber* Ag dressing, and UrgoClean Ag dressing. Each dressing was applied to a simulated wound assembly containing biofilm-gauze inoculated with *Klebsiella pneumoniae* and methicillin-resistant *Staphylococcus aureus* (MRSA). Each biofilm-inoculated gauze was incubated at 35±3^º^C for 6, 24, 48 and 72 hours. Enumeration of surviving biofilm bacteria at each time point was performed in triplicate for each test dressing and its equivalent control.

Results

Aquacel^®^ Ag+ Extra™ dressing was observed to reduce the biofilm population within 24 hours with a >4 log_10_ kill observed for *K. pneumoniae* and >6 log_10_ for MRSA from an initial biofilm challenge of 4.16×10^9^ CFU/mL. This kill rate was sustained for the duration of the challenge period, with Aquacel^®^ Ag+ Extra™ dressing reducing the biofilm population to non-detectable levels (<30 Colony Forming Units (CFU) per test) by 72 hours for *K. pneumoniae* and by 48 hours for MRSA. KerraContact^®^ Ag dressing demonstrated an initial reduction at 6 hours of ~2 log_10_ in both *K. pneumoniae* and MRSA. Durafiber* Ag dressing exhibited a slight, gradual reduction in biofilm population over the course of the test period, reducing each challenge organism by ~2.5 log_10_ by 72 hours. UrgoClean Ag was shown to have little to no impact on the dual-species biofilm with levels remaining similar or greater than that recovered prior to dressing application. The no-dressing biofilm-colonised gauze control demonstrated that the biofilm bacteria remained viable throughout the test period and species population proportionality was maintained.

Conclusion

Using a dual-species simulated wound biofilm model comprising the pathogens *K. pneumoniae* and MRSA, Aquacel^®^ Ag+ Extra™ dressing demonstrated significantly greater antibiofilm activity than the other silver-containing dressings. The enhanced antibiofilm activity of Aquacel^®^ Ag+ Extra™ dressing in this study may be attributed to the additional antibiofilm agents, ethylenediaminetetraacetic acid and benzethonium chloride, contained within the dressing.

## Introduction

Hard-to-heal wounds pose a significant clinical challenge [1] due to their association with reduced patient quality of life and significant economic burden [2,3]. Studies have shown that microorganisms found within hard-to-heal wounds frequently reside in biofilms [4], which are often polymicrobial in nature [5]. The polymicrobial nature of biofilm increases infection virulence and tolerance to antimicrobial treatments [6-8]. Therefore, antimicrobial wound dressings should ideally be effective not only against planktonic microorganisms but also against microorganisms within complex biofilm communities.

Ionic silver is a broad-spectrum topical antiseptic agent that is widely used in wound dressings to manage wound bioburden. Several studies have described the assessment of wound dressings using* in vitro* biofilm models. Suleman et al. (2020) evaluated the antibiofilm activity of silver-containing gelling fibre wound dressings using adapted and internally validated standard *in vitro* biofilm test methods, using both single and multispecies Centers for Disease Control and Prevention (CDC) and colony drip-flow reactor (CDFR) biofilm models [9]. An *in vitro* model designed to simulate a biofilm-colonised wound, using gauze-attached biofilm on a simulated moist wound bed with simulated peri-wound skin surrounding, has also been utilised to assess the antibiofilm activity of wound dressings [10,11]. However, the use of the model in published literature has been limited to single-species biofilm assessment [10,11]. The aim of this study was to expand on the biofilm gauze model, and develop a dual-species simulated wound biofilm model to evaluate the effectiveness of different silver-containing antimicrobial dressings.

Some of the results captured in this article were previously presented as a meeting abstract at the 2023 Wounds UK Annual Conference from 6-8 November 2023, the European Wound Management Association (EWMA) Annual Conference from 1-3 May 2024, and the Symposium on Advanced Wound Care (SAWC) Spring Annual Conference from 14-18 May 2024.

## Materials and methods

Test dressings

Four silver-containing wound dressings were evaluated *in vitro*. These were Aquacel^®^ Ag+ Extra™ dressing (Convatec, Deeside, UK), consisting of carboxymethylcellulose fibres containing ionic silver, ethylenediaminetetraacetic acid (EDTA) and benzethonium chloride; KerraContact^®^ Ag dressing (3M, Saint Paul, Minnesota, USA), consisting of non-adherent polyethylene mesh with a polyester core dressing containing silver oxysalts; Durafiber* Ag dressing (Smith & Nephew, Hull, UK), consisting of cellulose ethyl sulphonate fibres containing ionic silver; and UrgoClean Ag dressing (Urgo Medical Ltd., Loughborough, UK), consisting of polyacrylate (polyabsorbent) fibres with acrylic core containing silver sulphate.

Biofilm model

Separate suspensions of each challenge organism, *Klebsiella pneumoniae* (NCTC 9156) and community-associated methicillin-resistant *Staphylococcus aureus* ATCC^®^ BAA-1556™ (MRSA; USA300; HPA Reference: H045260142), were prepared in Maximum Recovery Diluent (MRD; Neogen Corporation, Lansing, MI, USA) and adjusted to yield a concentration of approximately 1x10^8^ colony forming units (CFU)/mL. These suspensions were further diluted in sterile 100 mL Duran bottles containing 9.9 mL of 50:50 Tryptone Soy Broth (TSB; Neogen)/Foetal Bovine Serum (FBS; Biowest SAS, Nuaillé, France) by adding 0.1 mL of the dual-species bacterial suspension (containing 0.02 mL of *K. pneumoniae *and 0.08 mL of MRSA) giving approximately a 1x10^6^ CFU/mL bacterial inoculum in total.

Samples of a 44 mm-diameter sterile knitted viscose gauze (N-A^®^ Gauze, 3M, Bracknell, UK) were aseptically prepared and transferred into the Duran bottles containing the inoculation medium. These were then placed in a shaking incubator for 24 hours set at 35°C (± 3°C) and 150 rpm. Following incubation, gauze samples were washed in 0.85% saline (Oxoid, Basingstoke, Hampshire, UK) (2 x 100 mL) to remove planktonic and loosely attached bacteria, then a sterile biopsy punch was used to cut the biofilm-colonised gauze substrates to a uniform size (35 mm in diameter). A total viable count (TVC) was then performed on washed, cut samples of the gauze substrate to confirm the level of biofilm (T_0hr_ count).

**Figure 1:**
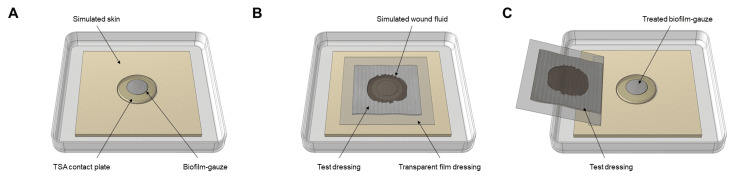
Test setup schematic (A) 24-hour biofilm grown on gauze is placed on an agar contact plate within the wound assembly. (B) Test dressing is applied and hydrated with simulated wound fluid. (C) Following incubation the test dressing is peeled back and the gauze-biofilm removed for total viable count processing. TSA: Tryptone Soy Agar

Figure 1 shows schematic of the simulated wound assembly set up. A series of Tryptone Soy Agar (TSA; Neogen) + 0.65% Agar Bacteriological (AB; Oxoid) contact plates were inserted into the centre of separate simulated wound assemblies (Figure 1A). Biofilm-colonised gauzes were then individually transferred onto each TSA + 0.65% AB contact plate to simulate a biofilm-colonised wound bed (n=3 per dressing for each time point assessed). Test dressings (10 x 10 cm samples) were applied to the simulated biofilm-colonised wound bed, hydrated with 8 mL of simulated wound fluid (SWF) (50:50 v/v MRD [Neogen]: FBS [Biowest]) and covered with a film dressing (Tegaderm™ Film, 3M Bracknell, UK) (Figure 1B). A biofilm-colonised gauze without a dressing applied was included as a control to monitor biofilm viability over the course of the challenge period (n=1 for each time point and each dressing type assessed).

Assessment of the test dressings

Following incubation at 35°C (± 3°C) for 6, 24, 48 or 72 hours, three simulated biofilm-colonised wound assemblies for each test dressing and one control were tested. Testing consisted of removing the test dressings (Figure 1C) and transferring the biofilm-colonised gauzes into separate stomacher bags containing 30 mL Dey-Engley Neutralising Broth (DENB; Neogen) to neutralise any residual antimicrobial activity. Each bag was then homogenised, using a laboratory stomacher, for 4 minutes to release biofilm bacteria from the gauze such that total viable counts (TVCs) could be performed on the resultant bacterial suspensions, to establish numbers remaining. TVCs were performed on the resultant bacterial suspension by diluting 1 in 10 dilution in DENB multiple times, then appropriate dilutions were plated onto pre-dried TSA and were incubated at 35 ±3^o^C for at least 48 hours. Following incubation, colonies on the most appropriate dilutions were counted and the bacterial number remaining on the gauze was calculated (CFU/gauze). Two-sample t-tests were conducted on results between test dressings using Microsoft Excel.

Confocal laser scanning microscopy

The dual-species biofilms were visualised under a confocal laser scanning microscope (CLSM; LSM800 with Airyscan, Zeiss, Germany). To ensure consistent biofilm was present, n=3 biofilm-colonised gauzes were imaged in three locations. Each of the three locations was cut to a 1 x 1 cm sample and bacteria within the biofilm were fluorescently tagged with 200 µL BacLight^®^ Live/Dead stain™ (Molecular Probes, Invitrogen, USA), prepared as stated by the manufacturer. This stain contains Syto 9 (green) for live bacteria and propidium iodide (red) for dead bacteria. In addition, 200 µL of DAPI (4’6-diamidino-2-phenylinode; Thermo Scientific, Waltham, MA, USA) stain was added to visualise extracellular DNA (eDNA) within the biofilm extracellular polymeric substances (EPS). Samples were left in darkness for 10 minutes prior to imaging, which was performed using x 40 objective lens with 488 nm and 630 nm lasers to excite the fluorophores within the BacLight^®^ Live/Dead stain™ and DAPI stain.

## Results

**Figure 2:**
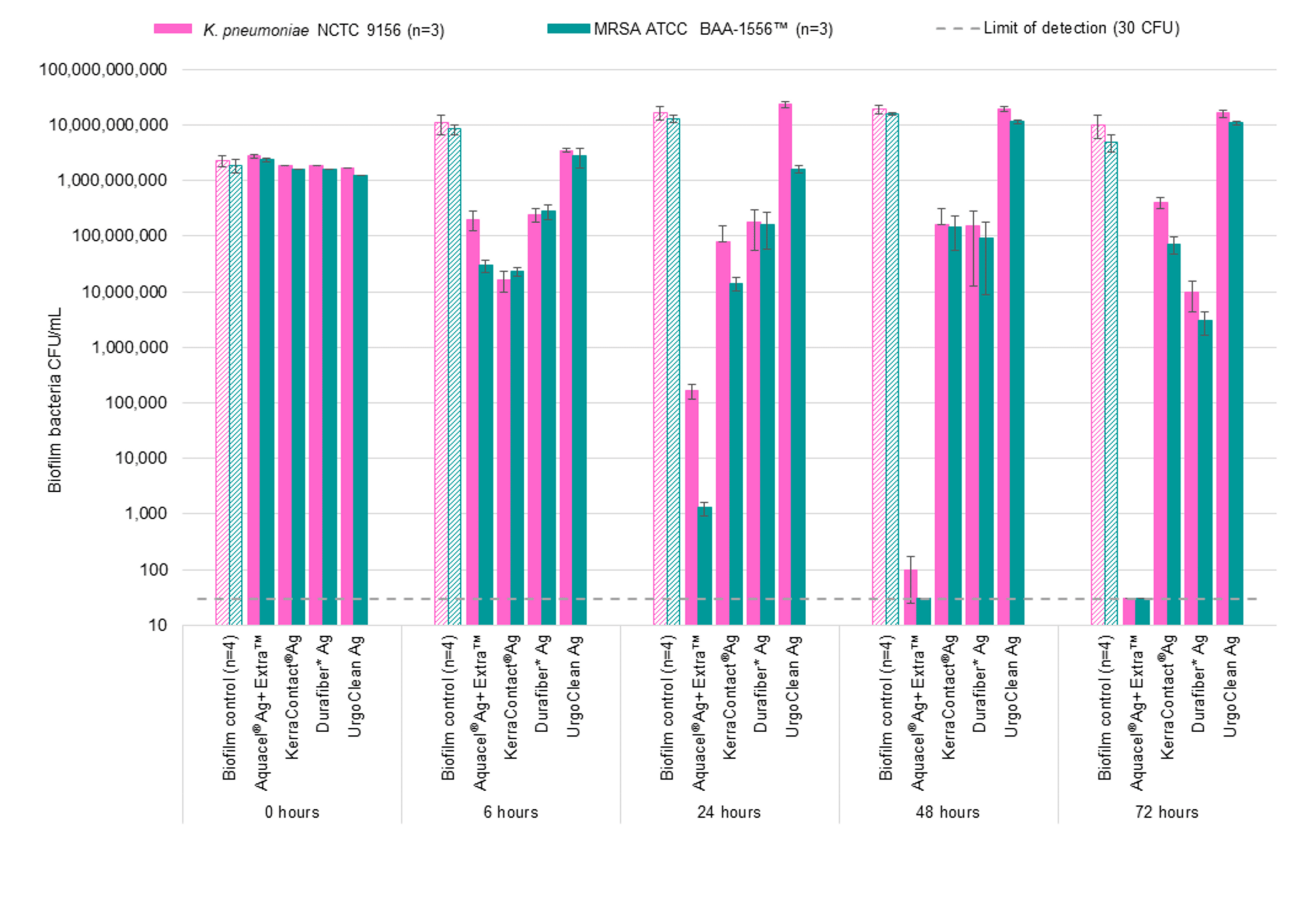
Antibiofilm activity of Aquacel® Ag+ Extra™, KerraContact® Ag, Durafiber* Ag and UrgoClean Ag dressings against a 24-hour dual-species biofilm (challenge organisms shown individually) of K. pneumoniae and MRSA over a 72-hour test period. The data has been normalized by subtracting the limit of detection (30 CFU). MRSA: Methicillin-resistant Staphylococcus aureus

**Figure 3:**
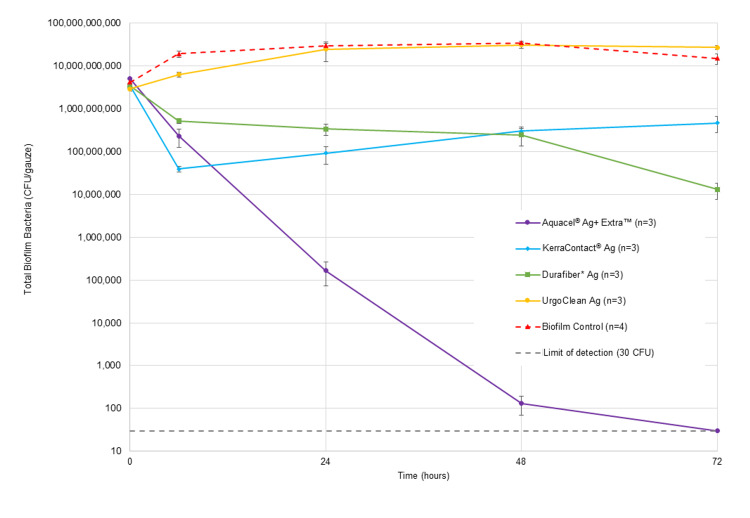
Antibiofilm activity of Aquacel® Ag+ Extra™, KerraContact® Ag, Durafiber* Ag and UrgoClean Ag dressings against a dual-species biofilm (total population biofilm) of K. pneumoniae and MRSA over a 72-hour test period MRSA: Methicillin-resistant Staphylococcus aureus

**Figure 4:**
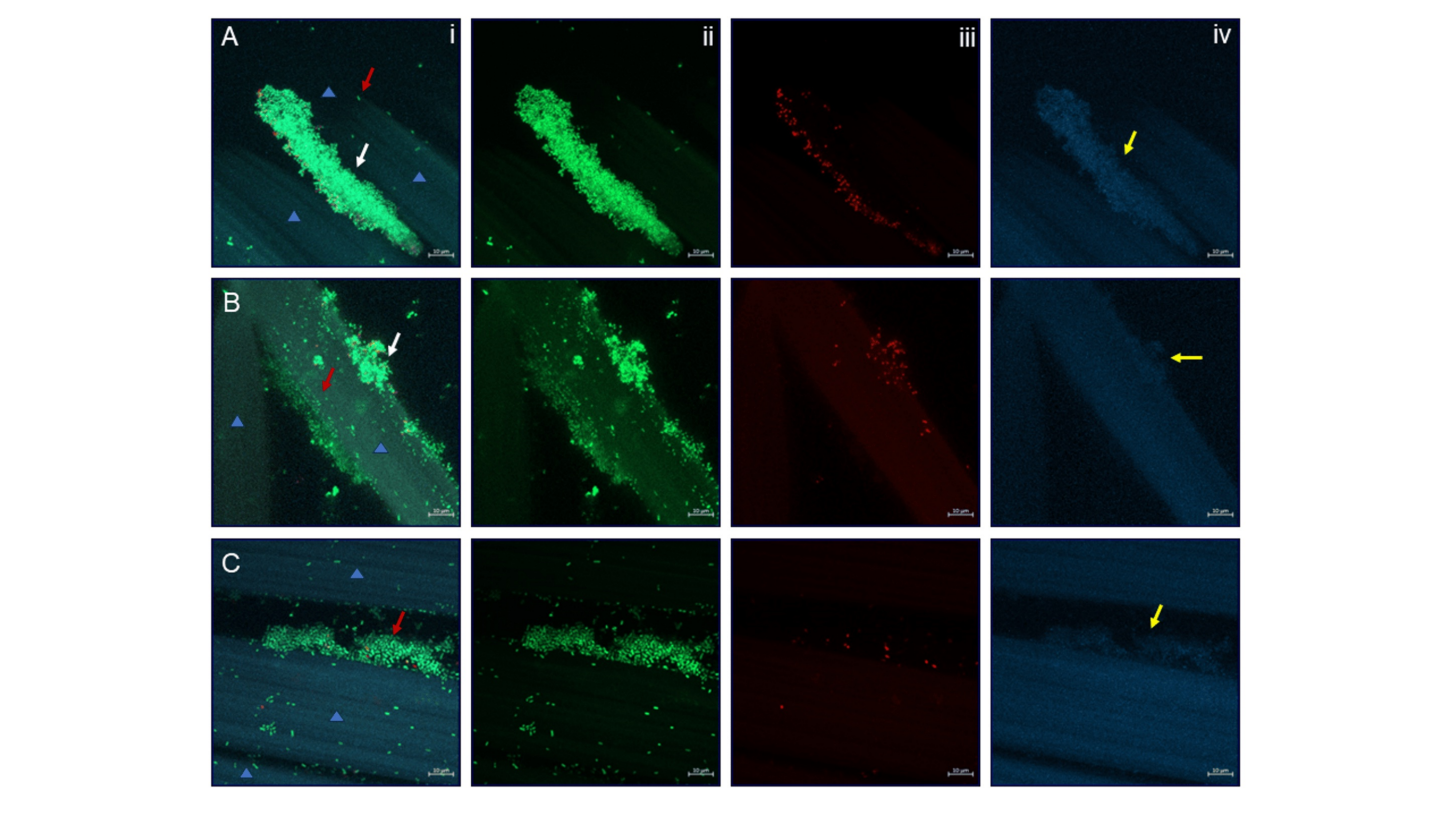
(A-C) Representative images from three separate biofilm colonised gauzes Dual-species biofilms were grown for 24 hours on gauze (gauze fibres indicated by blue triangles). Each biofilm-colonised gauze, A-C, is shown in four different ways: (column i) all stains combined; (ii) green (Syto 9) = live bacteria; (iii) red (propidium iodide) = dead bacteria; (iv) blue (DAPI) = eDNA. All gauze-biofilms contained areas of both MRSA (white arrows) and *K. pneumoniae* (red arrows) and eDNA (yellow arrows) before the application of test dressings.

During method development, initial inoculums of the two bacteria were varied so as to result in a similar number of each bacterial species in the final 24-hour biofilm before the dressings were applied (biofilm control at 0 hours; Figure 2). At the point of 24-hour growth, the dual-species biofilm contained total bacterial numbers of between ~2x10^9^ and ~5x10^9^ CFU/gauze (Figure 3). Bacterial numbers were also reflected in the microscopy images: representative images of the 24-hour dual-species biofilms are shown in Figure 4. The bacteria were fluorescently tagged green if alive (Figure 4, column ii) and red if dead (Figure 4, iii), while blue revealed eDNA within the biofilm EPS matrix (Figure 4, iv). Biofilm microcolonies were observed for MRSA (Figure 4A), for both species (Figure 4B), and for *K. pneumoniae *(Figure 4C). The presence of eDNA within these microcolonies confirmed that the bacterial cells were in the biofilm phenotype (Figure 4A-4C, column iv).

Aquacel^®^ Ag+ Extra™ dressing was observed to reduce the biofilm population within 6 hours of application for both bacterial species in the biofilm by ~1 log_10_ (Figures 2, 3). Within 24 hours, a >4 log_10_ kill was observed for *K. pneumoniae* and >6 log_10_ for MRSA from an initial total biofilm challenge of ~1x10^8^ CFU/mL (Figure 3). This kill rate was sustained for the duration of the challenge period, with Aquacel^®^ Ag+ Extra™ dressing reducing the biofilm population to non-detectable levels (<30 CFU per test) by 72 hours for *K. pneumoniae* and 48 hours for MRSA (Figure 2). Therefore, Aquacel^®^ Ag+ Extra™ dressing reduced a dual-species biofilm challenge containing both *K. pneumoniae *and MRSA to below the limit of detection within 72 hours of application (Figure 3).

KerraContact^®^ Ag dressing demonstrated an initial reduction at 6 hours of ~2 log_10_ (100-fold reduction) in both *K. pneumoniae* and MRSA (Figure 2). However, this dressing was unable to further reduce the biofilm population after this time, with counts at subsequent time points being similar or greater than that at 6 hours for both organisms in the biofilm (Figures 2, 3).

Durafiber* Ag dressing exhibited a slight, gradual reduction in biofilm population over the course of the test period (Figure 3). However, by 72 hours only a ~2.5 log_10_ decrease in each challenge organism population was achieved (Figure 2).

UrgoClean Ag dressing was shown to have little to no impact on the dual-species biofilm, with levels remaining similar or greater than that recovered prior to dressing application (T_0hr_ count; Figures 2, 3). At 72 hours, the total biofilm recovered from biofilm-colonised gauze with this dressing applied was higher than that of the no-dressing biofilm control (Figure 3).

The no-dressing biofilm-colonised gauze samples demonstrated that the biofilm bacteria remained viable throughout the test period (Figures 2, 3) and that species population proportionality was maintained (Figures 2, 3).

In two-sample t-tests, Aquacel^®^ Ag+ Extra™ dressing resulted in significantly lower survival of both *K. pneumoniae* and MRSA biofilm at all time points (6, 24, 48 and 72 hours) compared to KerraContact^®^ Ag and UrgoClean Ag (all p<0.05), with the exception of MRSA at 6 hours for KerraContact^®^ Ag. Aquacel^®^ Ag+ Extra™ resulted in significantly lower survival of MRSA biofilm at 6 hours, and both *K. pneumoniae *and MRSA biofilm at 72 hours, compared to Durafiber* Ag (p<0.05). Aquacel^®^ Ag+ Extra™ dressing resulted in significantly lower total biofilm counts at all time points compared to all other test dressings (p<0.05), with the exception of 24 hours and 48 hours for Durafiber* Ag.

## Discussion

The clinical complexity of wound biofilm is challenging to replicate within the laboratory environment. While complex, single-species models have been used in the past to assess the antibiofilm properties of silver-containing dressings [10], including the use of antibiotic-resistant pathogens [11], these models do not fully replicate the polymicrobial nature of most hard-to-heal wounds. This study expanded on the gauze-attached biofilm model [10,11], with a dual-species biofilm containing *K. pneumoniae* and an antibiotic-resistant strain of *S. aureus*. The dual-species biofilm contained high and similar counts of both challenge organisms, with the combined total being similar to the previous single-species studies [10,11]. The controls demonstrated that the dual-species biofilm remained viable throughout the study - a total of 72 hours - and maintained species proportionality of *K. pneumoniae *and MRSA.

Obtaining proportionality between species within dual-species and polymicrobial biofilms in the laboratory environment can be difficult, and in other studies, researchers have struggled to keep the numbers of each species high, due to their competitive nature [9]. Microorganisms have been shown to inhibit each other’s ability to attach and form biofilms, and can also initiate the dispersal of species from polymicrobial biofilms [12]. Polymicrobial biofilms can develop spatially in different ways [13], and in the present study, the biofilm, possibly due to its age (24 hours), demonstrated proximate but individual areas of the two bacterial species without coaggregation (mixing within the same location). Future testing using microscopy could be performed to evaluate this observation further, assess antibiofilm dressing activity against various polymicrobial biofilms, and examine whether the different spatial distributions of microorganisms, i.e., separate microcolonies, coaggregation or layered distribution, have an effect.

While all dressings assessed in the study were silver-containing dressings, their antibiofilm activities differed, with Aquacel^®^ Ag+ Extra™ showing far greater activity than the other dressings, which in some cases reached statistical significance. The observed differences in antibiofilm properties cannot be accounted for by the levels of silver content of the dressings. Despite having differing silver concentrations of 0.17 mg/cm^2^ (1.2% w/w) as ionic silver (Ag^+^ ions: the primary antimicrobial form of silver) for Aquacel^®^ Ag+ Extra™ [14], approximately 0.4 mg/cm^2^ (4.5% w/w) as silver oxysalts (ionic silver including short-lived, highly active Ag^3+^ ions) for KerraContact^®^ Ag [15,16], approximately 3.5% w/w as silver sulphate (a silver compound, Ag_2_SO_4_, which dissociates into Ag^+^ ions) for UrgoClean Ag [17], no direct correlation between silver content and antibiofilm efficacy was evident. The silver content for Durafiber* Ag was not found to be publicly available. In addition, all the dressings assessed in the study claimed to have antibiofilm properties [10,15,18], except for Durafiber* Ag. The results suggest that the greater antibiofilm activity of Aquacel^®^ Ag+ Extra™ compared with the other dressings, may be attributed to the additional antibiofilm agents within the dressing: EDTA (a chelator of divalent metal cations, such as calcium (Ca^2+^), iron (Fe^2+^) and magnesium (Mg^2+^), which provide strength to biofilm EPS matrices [10,19]) and benzethonium chloride (a surfactant which acts in synergy with EDTA and ionic silver) [10,14,19].

Due to the increasing problem of antibiotic-resistant bacteria and the subsequent requirement for enhanced antibiotic stewardship, there is a need for alternative strategies to reduce the spread of antibiotic resistance [20], and for laboratory models to assess antibiotic-resistant pathogens. In the present study, a specifically designed antibiofilm dressing, Aquacel^®^ Ag+ Extra™, was shown to be the most effective against a dual-species biofilm containing an antibiotic-resistant strain of pathogen (MRSA). The results suggest that Aquacel^®^ Ag+ Extra™ may provide a potential strategy against biofilms containing antibiotic-resistant bacteria in wounds. The CLSM imaging carried out prior to dressing application in this study established the presence of eDNA within the attached and aggregate biofilms, indicating the presence of EPS. EPS aids biofilm structurally, but can also enable horizontal gene transfer between bacteria, which may include the transfer of antibiotic resistance genes [21]. Therefore, as in the present study, using antibiotic-resistant strains in polymicrobial biofilm, adds further complexity to laboratory models for the assessment of the antibiofilm performance of wound dressings.

There are several limitations to the current study. *In vitro* models cannot replicate real wound biofilm and it is unclear how the findings translate to clinical practice. This study investigated a dual-species model; however, clinical wound biofilm is usually polymicrobial [22] and may include multiple species of bacteria and fungi [23]. Future work could progress the described biofilm model to include additional species and different combinations of microorganisms which may be found in clinical wound biofilms. Suleman et al. (2020) described the use of standard adapted polymicrobial biofilm models using two bacterial species, *S. aureus* and *Pseudomonas aeruginosa*, along with a fungus, *Candida albicans* [9]. Additionally, the model could be utilised to investigate other types of antibiofilm dressings and products: a similar single-species gauze biofilm model has been validated and used to assess the antibiofilm performance of a wound debridement gel compared to other wound debridement methods [24,25].

## Conclusions

This simulated wound model accounted for the polymicrobial nature and surface-associated/aggregated biofilm phenotype of microorganisms often found in hard-to-heal wounds. The study demonstrated that not all silver-containing dressings are equally effective against this complex microbial phenotype, with Aquacel^®^ Ag+ Extra™ demonstrating significantly greater antimicrobial activity than the other silver-containing dressings. The enhanced antimicrobial activity of Aquacel^®^ Ag+ Extra™ in this study may be attributed to the additional agents (EDTA and benzethonium chloride) contained within the dressing, which help the silver perform effectively against biofilm microorganisms.
